# Case Report: A Re-Positive Case of SARS-CoV-2 Associated With Glaucoma

**DOI:** 10.3389/fimmu.2021.701295

**Published:** 2021-07-28

**Authors:** Xiaoli Zhou, Ya-Na Zhou, Ashaq Ali, Cuiqin Liang, Zhiqin Ye, Xiaomin Chen, Qing Zhang, Lihua Deng, Xinyi Sun, Qian Zhang, Jihong Luo, Wei Li, Kun Zhou, Shanshan Cao, Xiaowei Zhang, Xiao-Dong Li, Xian-En Zhang, Zongqiang Cui, Dong Men

**Affiliations:** ^1^Hubei Province Hospital of Traditional Chinese Medicine, Wuhan, China; ^2^State Key Laboratory of Virology, Wuhan Institute of Virology, Center for Biosafety Mega-Science, Chinese Academy of Sciences, Wuhan, China; ^3^University of Chinese Academy of Sciences, Beijing, China; ^4^National Laboratory of Biomacromolecules, CAS Center for Excellence in Biomacromolecules, Institute of Biophysics, Chinese Academy of Sciences, Beijing, China; ^5^First Clinical College, Hubei University of Chinese Medicine, Wuhan, China

**Keywords:** aqueous humor, COVID-19, glaucoma, immunostaining, SARS-CoV-2

## Abstract

The current pandemic of coronavirus disease 2019 (COVID-19), caused by severe acute respiratory syndrome coronavirus 2 (SARS-CoV-2), has already become a global threat to the human population. Infection with SARS-CoV-2 leads to a wide spectrum of clinical manifestations. Ocular abnormalities have been reported in association with COVID-19, but the nature of the impairments was not specified. Here, we report a case of a female patient diagnosed with glaucoma on re-hospitalization for ocular complications two months after being discharged from the hospital upon recovery from COVID-19. Meanwhile, the patient was found re-positive for SARS-CoV-2 in the upper respiratory tract. The infection was also diagnosed in the aqueous humor through immunostaining with antibodies against the N protein and S protein of SARS-CoV-2. Considering the eye is an immune-privileged site, we speculate that SARS-CoV-2 survived in the eye and resulted in the patient testing re-positive for SARS-CoV-2.

## Introduction

Coronavirus disease 2019 (COVID-19), caused by severe acute respiratory syndrome coronavirus 2 (SARS-CoV-2), has been identified among patients in China since December 2019. The infection has rapidly spread worldwide (1). As a newly emerging infectious disease, details regarding COVID-19 are not yet fully elucidated. SARS-CoV-2 causes substantial pulmonary disease and is associated with detrimental effects on several other processes, such as cardiovascular, gastrointestinal, hematologic, renal, endocrinologic, dermatologic, neurologic, and ophthalmologic. At the same time, diverse abnormal immune responses of the human body towards SARS-CoV-2 infection have been observed, such as early waning of protective immune responses with a rapid reduction in IgG/IgM and neutralizing antibody levels, during the early phase of convalescence (2) or no responses at all. Recently, the appearance of reinfection (3), re-positivity, and long-term positivity of SARS-CoV-2 (4) has attracted more attention, as SARS-CoV-2 may be evading the immune system to make these patients a potential source of infection. Coronavirus has been previously described to be associated with human conjunctivitis (5). While there is no direct proof that replication of SARS-CoV-1 results in conjunctivitis and other ocular diseases, studies have highlighted the eye as a possible site for transmitting viruses (6). Previous studies confirmed the crucial role of CD147 in promoting SARS-CoV-2 invasion into host cells and CD147 is present in tears, aqueous humor, and vitreous fluids (7). A study (8) with 7% of patients presenting viral RNA in their conjunctival secretion has further emphasized the possibility of SARS-CoV-2 ocular transmission. The transmission of SARS-CoV-2 through the eye has been suspected. Moreover, the unusual COVID-19 cases, reports of re-infection or secondary infection events by SARS-CoV-2 with few shreds of evidence indicating ocular transmission need more attention

### Case Description

Here we report a case of a 66 year-old woman admitted to the Hubei provincial hospital of Traditional Chinese Medicine (Wuhan, Hubei) with symptoms such as fever, sore throat, cough, and muscle pain on January 21, 2020. The laboratory examinations revealed an elevated level of C-reactive protein, decreased lymphocyte counts, and increased neutrophil counts. Thoracic computed tomography (CT) scan showed multiple ground-glass opacities in the bilateral upper lobes of the lungs, indicating the possibility of viral pneumonia. Then oropharyngeal swab specimen from the upper respiratory tract was obtained, and the nucleic acid tests for SARS-CoV-2 were positive. This patient was diagnosed as SARS-CoV-2 positive and was hospitalized till February 13, 2020 with commonly recommended medication. Upon recovery from COVID-19, the patient displayed sight darkness and a spiral visual field in her left eye. An ocular swab and tear samples were collected from both eyes, and the SARS-CoV-2 RT-PCR test result was negative. Following the hospital recommended criterion, the patient was discharged after having tested negative twice for the SARS-CoV-2 RNA.

**Figure 1:**
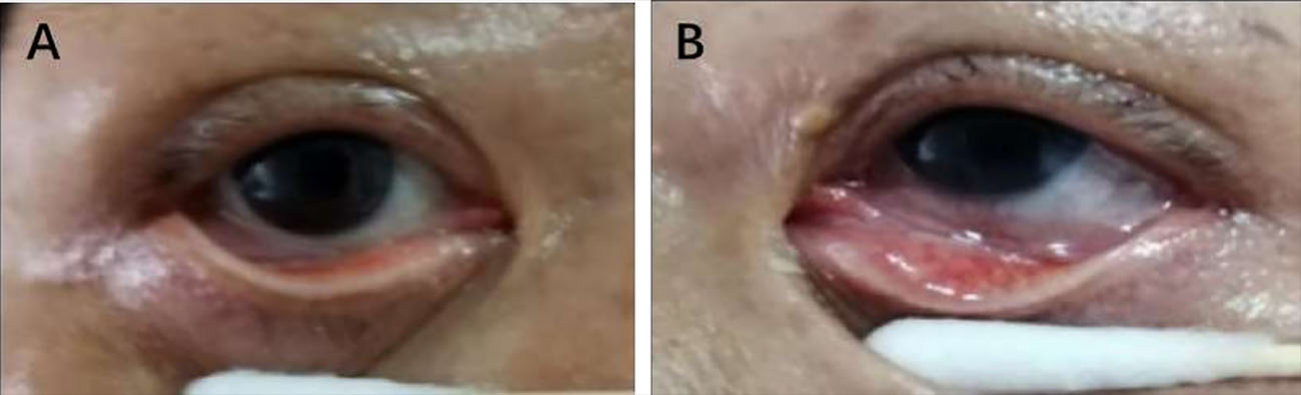
Slit-lamp photographs of the patient’s eyes. Conjunctival congestion and conjunctival follicles in the left eye **(B)** and the right eye was normal **(A)**.

However, the patient was admitted to the hospital again on April 22, 2020 after developing more complex ocular conditions. Upon reassessment, the SARS-CoV-2 RNA test was also found positive thereby complicating the case. Oropharyngeal and conjunctival swabs were collected, and SARS-CoV-2 RNA was detected in both throat and left eye. The patient experienced symptoms of glaucoma such as halos around lights, blurred vision, and progressive loss of peripheral or side vision. The slit-lamp evaluation showed epiphora, conjunctival congestion, and inferior palpebral and conjunctival follicles in the left eye (**Figure 1**). An ophthalmic test revealed a visual acuity of 20/200 in her left eye. The intraocular pressure (IOP) of the right eye and left eye was 18.1 mmHg and 48 mmHg, respectively. The patient was diagnosed with unilateral glaucoma.

On re-hospitalization, the patient’s IgM was found positive, slightly higher than the cutoff value, indicating that the body still had an active response to the infection (**Table 1**). In contrast, IgG antibodies were observed to be at a higher level against SARS-CoV-2. Aiming to evaluate the patient’s immune system functionality, the patient’s inactivated serum sample was analyzed using a plaque reduction neutralization test (PRNT). With the value for nCoV T-3 = 1068.01, RBD IgG = 450.07, Surrogate Virus Neutralization Test (SVNT) 2-1 = 36.11, SVNT3 = 35.6 and PRNT_50_ = 89. Therefore, we observed that the patient’s serum neutralizing antibodies protected her against infection, to a certain extent, by inhibiting SARS-CoV-2, thereby indicating that the patient’s immune system was functioning normally.

**Table 1:** RT-qPCR results and antibodies titer calculated for four consecutive weeks.

Type of samples	Source of samples	Date of sample collection			
		05-07	05-14	05-21	05-28
	Throat swab	**41.29**	–	–	–
Swab samples					
	Saliva	–	**36.50**	–	–
	Tears	–	–	–	–
	Anal	–	–	–	–
Tissue sample					
	Sputum	–	–	–	–
	Aqueous humor	NA	NA	NA	**35.00**
Serum samples	IgG U/µL	**76.84**	**63.74**	**65.03**	**67.68**
	IgM U/µL	**10.19**	**11.72**	**9.12**	**9.29**

NA, Not Applicable/samples not taken. Cutoff value for IgG =10 U/µL, and IgM = 1 U/µL

**Figure 2:**
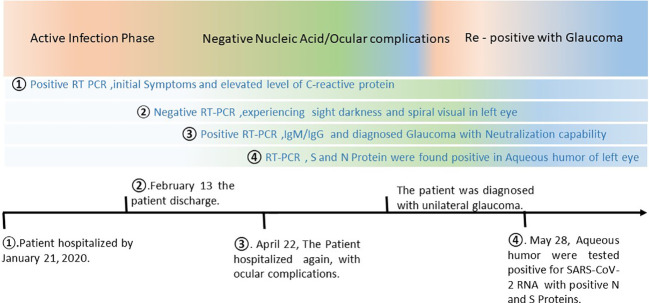
Timeline of the COVID-19 infection, development of glaucoma, and recurrence SARS-CoV-2. On January 21, 2020, the patient was presented to the Hubei provincial hospital of Traditional Chinese Medicine with initial COVID-19 signs and symptoms and eventually confirmed to be positive for SARS-CoV-2 infection using RT-PCR. After normal recommended medication, the patient was discharged from the hospital on February 13, when declared negative with RT-PCR twice as per hospital criterion. However, the patient was experiencing sight darkness and spiral visual field in her left eye. On April 22, 2020, the patient was re-hospitalized displaying more severe unilateral ocular complications. With an extensive investigation the patient was found re-positive for SARS-CoV-2 with neutralizing antibodies. The ocular complication resulted in unilateral glaucoma. The investigation team further examined the patient closely, and the immunocytochemical study of both eyes was conducted on May 28, 2020. SARS-CoV-2 spike (S) or Nucleocapsid (N) protein were detected in aqueous humor of the left eye only.

Next, we tried to investigate how the virus could still linger in the body, despite the presence of neutralizing antibodies, which can inhibit the progression of SARS-CoV-2 infection. We further examined the viral load of the upper respiratory tract (throat swab and saliva), digestive tract (anal swabs), and eyes (tears) to determine whether the virus was still present in these locations. As shown in **Table 1**, the RNA detection results were found weakly positive in the upper respiratory tract and negative in the digestive tract and tear samples. These results indicated that the virus was not present in the lower respiratory tract and digestive tract, and only a low viral load was present in the upper respiratory tract. Together, these results suggested that SARS-CoV-2 was avoiding the immune clearance and still harbored at some locations in the patient’s body. The timeline depiction of the current case report (**Figure 2**).

**Figure 3:**
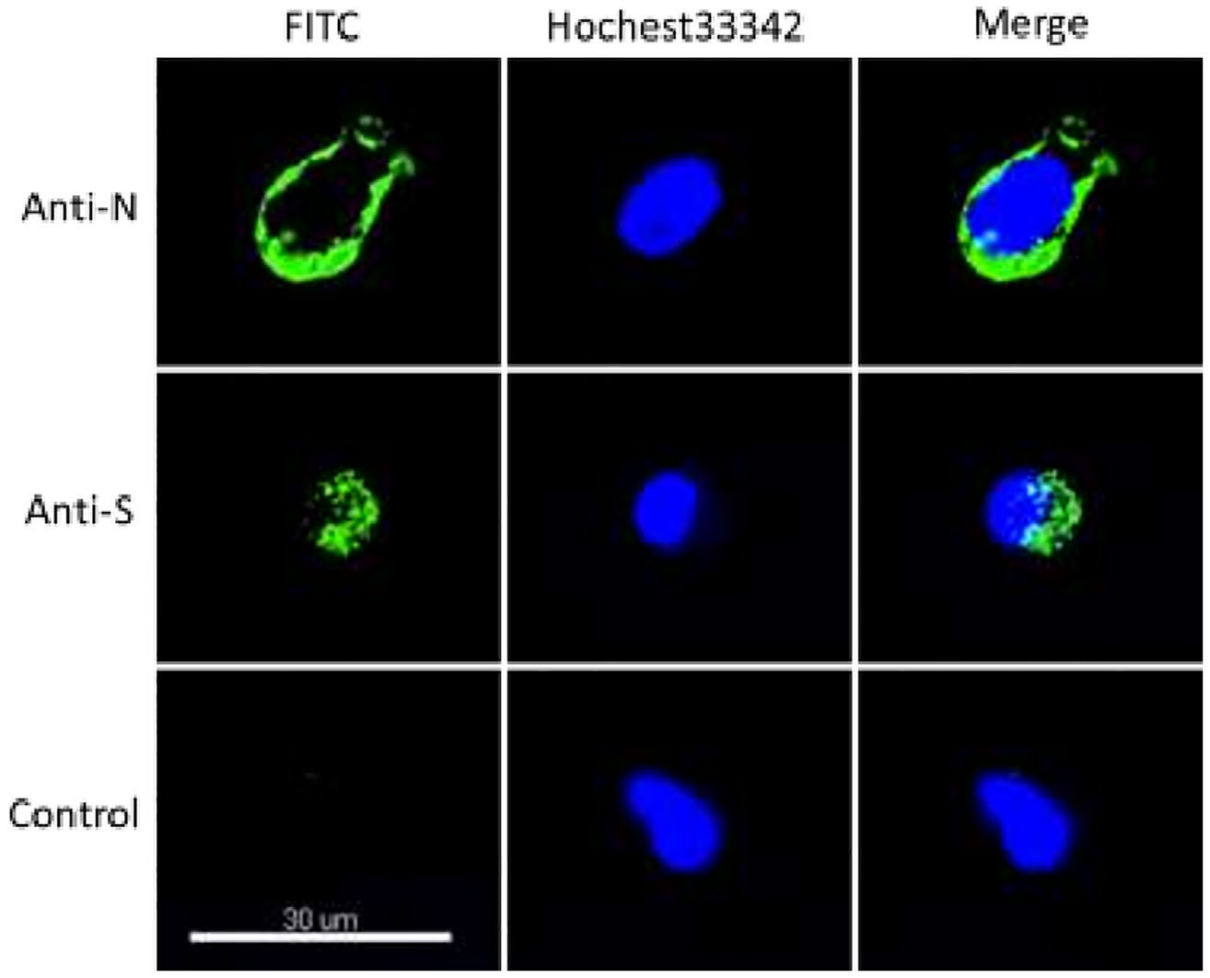
SARS-CoV-2 was detected using immunofluorescence in the aqueous humor. Cells were fixed and incubated with the antibodies of SARS-CoV-2 spike (S) protein, nucleocapsid (N), respectively. This picture shows SARS-CoV-2 was detected in the aqueous humor of the left eye.

Fortunately, the patient was subjected to surgery to cure glaucoma. Thus aliquots, of the aqueous humor in the anterior chamber of the patient’s left eye were obtained. We were able to acquire a fraction of the sample and therefore could explore the association of SARS-CoV-2 with glaucoma. Moreover, we wanted to examine the presence of SARS-CoV-2 behind blood eye barrier or in the immune-privileged site of the eyes, to explore the possibility of ocular transmission of this virus. Eventually, the aqueous humor tested positive for SARS-CoV-2 RNA on May 28, 2020. The cycle threshold value for the aqueous humor was approximately 35. Therefore, to confirm the presence of SARS-CoV-2, the cells from the aqueous humor were stained with the antibody against SARS-CoV-2. For cytology studies, 10 μL of aqueous humor was spotted on poly-L-lysine-coated glass slides. The immunocytochemical study was performed using antibodies against the SARS-CoV-2 spike (S) protein or nucleocapsid (N) protein. The N protein was found in the cytoplasm and the S protein gathered around the nucleus in the cells of the left eyes (**Figures 3** and **S2**), but not in the cells collected from the right eye (**Figure S1**). These results validated the latent SARS-CoV-2 that was found in the left eye of the patients. Considering that the eye is a part deprived of the immune system, SARS-CoV-2 entered into the eye’s interior through an unknown route and was able to avoid the immune clearance. The above results suggested that SARS-CoV-2 could infect immune-privileged sites to cause patients to test re-positive or remain positive for SARS-CoV-2 for a longer period of time.

## Diagnostic Assessment

### Clinical Studies

The patient, when admitted to Hubei provincial hospital of Traditional Chinese Medicine Wuhan, Hubei, for the first time was treated as a regular COVID-19 patient. However, upon re-hospitalization, the patient was admitted to the Intensive Care Unit under the keen observation of ophthalmologists and infectious disease experts. In addition to COVID-19 treatment, the patient also received additional supportive treatment for ocular complications. The patient provided written informed consent for publication of the information presented in this case report.

### Diagnosis

#### RT-PCR

RT-PCR was performed for confirmation of SARS-CoV-2 in nasal, oropharyngeal, lachrymal, and aqueous humor samples, according to the established protocol by the Chinese Center for Disease Control (9). ORF1 ab and N gene of SARS-CoV-2 were detected with the 2019-nCoV nucleic acid detection kit (fluorescence PCR method) developed by DaAn Gene Co., Ltd. details are shown in supplementary information.

#### Immunocytochemical Study

Immunocytochemical analysis was carried out to investigate the expression of both N and S proteins in the aqueous humor cells. The commercially available primary antibodies (Anit-N, DA00027 and Anti-S, DA00005) were purchased from symray biopharma co. Ltd. Goat Anti-Human IgG Fc (FITC) (ab97224) was purchased from abcam Ltd. Hoechst 33342 was acquired from beyotime (C1022). Assays details are provided in supplementary files. The aqueous humor was collected from both eyes of the patient provided by Hubei provincial hospital.

#### Plaque Reduction Neutralization Test (PRNT)

The plaque reduction neutralization test was used to examine the patient’s serum sample for their neutralization capability against SARS-CoV-2. The PRNT was used as described previously with minor modifications (10). Details of the PRNT assays are provided in supplementary document.

## Discussion

The current case highlights the persistent behavior of COVID-19 infection by demonstrating provoked immune responses. Similarly, the weak positive PCR results of SARS-COV-2 RNA in the upper respiratory tract and negative results for the sputum samples and those from the digestive tracts indicated that the body’s immune system did not entirely eliminate the infection. However, the immunostaining experiment provides experimental evidence that SARS-CoV-2 infection occurs in the eyes, an immune-privileged site in the human body. Notably, the immunocytochemical study revealed the presence of SARS-COV-2 in the left eye only, making the current study noteworthy.

There are several reports about the COVID-19 infection found in the immune-privileged sites, for example, the detection of SARS-CoV-2 RNA and expression of the S and N proteins in the placenta of a COVID-19 pregnant woman (11), the direct cytolytic effect of SARS-CoV-2 on the pancreas (12), and its association with certain CNS complications (13). Previous experimental animal-based studies of the coronavirus infection, reported retinal diseases such as retinal vasculitis (14) and retinal degeneration and blood-retinal barrier breakdown (15) revealed the possibility of immune-privileged site infectivity by SARS-CoV-2. Furthermore, the ACE2 expression on the eye was reported (7) and also found to be associated with the treatment of glaucoma and uveitis (16). It was also reported that SARS-CoV-2RNA was detected on the ocular surface of 52 COVID-19 patients (17). Likewise, RNA sequencing analysis also revealed that the virus infected the ocular surface cells, especially the limbus. These reports support the finding of our current case study. Consequently, it shows that SARS-CoV-2 could infect the eyes and survive in immune privileged site of the eyes with the potential of causing infection. Our result further opens the question of the association of SARS-CoV-2 with glaucoma.

In the case presented, we strongly suspect that the immune-privileged site, the eye, must have caused the contraction of the SARS-CoV-2 infection and the episodic weak presence of SARS-CoV-2 resulting in COVID-19 recurrence. It also highlighted the importance of periodic follow-up of COVID-10 patients to avoid the development of a raised IOP that could lead to glaucoma. The current case illustrates that SARS-CoV-2 most likely evades immune clearance, thereby persistently proliferating and spreading. In conclusion, the recurrent COVID-19 cases demanded detailed investigations of associated conditions like glaucoma, revealing what is likely to be a series of patients testing re-positive.

Unfortunately, we still unable to describe the true nature of the current recurrence of SARS-CoV-2, that either SARS-CoV-2 survived in immune privileged sites is still infectious or not. Our findings are based on single case study, and currently as there are no new cases herein China. Therefore, we are unable to investigate further to divulge similar cases. However, the phenomena is a challenge for ophthalmologist as well as for the researchers and demanding further investigation to sort out the lingering effects of COVID infection and the recurrence of SARS-CoV-2.

## Data Availability Statement

The raw data supporting the conclusions of this article will be made available by the authors, without undue reservation.

## Ethics Statement

The studies involving human participants were reviewed and approved by Hubei Provincial Hospital of Traditional Chinese Medicine (Wuhan, Hubei). The patients/participants provided their written informed consent to participate in this study.

## Author Contributions

XZ, YZ, ZY, QingZ, JL, XC, LD, XS, and QianZ cared for the patient and extracted the data from the hospital. CL, KZ, AA, SC, XWZ, WL performed laboratory experiments. ZQC, AA contributed to writing of the case report. XZ, XD, and DM supervised the case. Written consent from the patient was obtained to publish this case report. All authors contributed to the article and approved the submitted version.

## Funding

This work was supported by the National Key Research and Development Program of China (Grant No. 2020YFC0861100). The Strategic Priority Research Program of the Chinese Academy of Sciences (XDB29050201), The Scientific Research Fund of Traditional Chinese Medicine of Hubei Health Commission (Grant No. ZY2021M035) and the Youth Innovation Promotion Association of CAS (Grant No. 2014308), and the Interdisciplinary Innovation Team of CAS.

## Conflict of Interest

The authors declare that the research was conducted in the absence of any commercial or financial relationships that could be construed as a potential conflict of interest.

## Publisher’s Note

All claims expressed in this article are solely those of the authors and do not necessarily represent those of their affiliated organizations, or those of the publisher, the editors and the reviewers. Any product that may be evaluated in this article, or claim that may be made by its manufacturer, is not guaranteed or endorsed by the publisher.
