# Supplementary material for: Case Report: A Re-Positive Case of SARS-CoV-2 Associated With Glaucoma
Source: Front Immunol. 2021 Jul 28;12:701295. doi: 10.3389/fimmu.2021.701295 (PMC8355891; doi:10.3389/fimmu.2021.701295)
Supplement: Supplementary file 1 [file DataSheet_1.pdf]

Supplementary Files

A re-positive case of SARS-CoV-2 associated with glaucoma

Xiao-Li Zhou[1†], Ya-na Zhou[1†], Ashaq Ali[2,4†], Cui-qin Liang [2†], Zhi-qin Ye[1], Kun Zhou[2,4], Qing Zhang[1], Ji-hong Luo[1], Xiao-Min Chen[1], Li-hua Deng[1], Xin-yi Sun[1], Qian Zhang[1], Shan-shan Cao[2], Xiao-wei Zhang[2], Wei Li[2], Xian-En Zhang[3,4*], Xiao-Dong Li[1,5*], Zong-Qiang Cui[2,4*], Dong Men[2,4*]

3.2.1 RT-PCR

The Nucleic Acid detection was carried out using Novel coronavirus 2019-nCoV nucleic acid detection kit (fluorescence PCR method) developed by DaAn Gene Co., Ltd. By targeting ORF1 ab and N gene of SARS-CoV-2.

Target 1（ORF1ab）:

（F）: CCCTGTGGGTTTTACACTTAA

（R）: ACGATTGTGCATCAGCTGA

（P）: 5'-FAM-CCGTCTGCGGTATGTGGAAAGGTTATGG-BHQ1-3'

Target 2（N）:

（F）: GGGGAACTTCTCCTGCTAGAAT

（R）: CAGACATTTTGCTCTCAAGCTG

（P）: 5'-FAM-TTGCTGCTGCTTGACAGATT-TAMRA-3'

3.2.2 Immunocytochemical study

Sample treatment: Almost 30μL of aqueous humor for each eye was collected. The samples were centrifuged at 500g for 5min, and the liquid was absorbed. The precipitations were fixed with 4% paraformaldehyde. SARS-CoV-2 spike (S) protein, nucleocapsid (N) protein from the lab of Prof. Men. Goat Anti-Human IgG Fc (FITC) were purchased from abcam ab97224. Hoechst 33342: from beyotime with catalogue number C1022 were used in the current assay.

Immunofluorescence analysis:

1. The precipitation in aqueous humor of both eyes were in two tubes, add 50μL 4% paraformaldehyde into each tube, suspended the cells slowly, and fixed them at room temperature for 30 minutes;
2. The fixative was removed after centrifugation at 500g for 5 minutes and then washed the precipitation with PBS for once；
3. After centrifugation at 500g for 5 minutes, most of the liquid was absorbed and about 60 μL of liquid was retained. Then the cells were slowly suspended equally to add to three confocal dishes. Make sure the cells were as evenly distributed as possible;
4. Put the confocal dishes in the oven at 50℃ for about 10 minutes;
5. After there were no liquid on the confocal dishes, 0.5%Triton-100 was added to operate the cells for 5 minutes at room temperature;
6. Discarded the liquid, washed confocal dishes with PBS for 3 minutes, gently;
7. 10% FBS was used to block the cells at 37℃ for 60 minutes;
8. The left and right eyes aqueous cells of three confocal dishes were incubated with the antibodies of SARS-CoV-2 spike (S) protein, nucleocapsid (N) protein or 1%FBS at 4℃ overnight, respectively. Both antibodies were diluted with 1%FBS at 1:500;
9. The primary antibodies were removed, then gently washed the confocal dishes with PBS three times for three minutes each time;
10. Remove the washing liquid, protect the environment from light, and add the fluorescently labeled secondary goat anti-human IgG Fc (FITC) antibodies diluted by 1:1000, acting at 37℃ for 60 minutes;
11. The second antibodies were removed, then gently washed the Confocal dishes with PBS three times for three minutes each time;
12. The nucleus was stained with Hoechst for 5 minutes at room temperature;
13. Gently washed with PBS three times for three minutes each time;
14. Observed under a fluorescence microscope.

The right eye aqueous humor was treated in the same way as the left eye aqueous humor. However, it is difficult to find the more cells on the confocal dishes under the fluorescence microscope. It is possible that the patient's right eye not injured as like left eye.

The results of aqueous humor in the right eye Fig S.1.

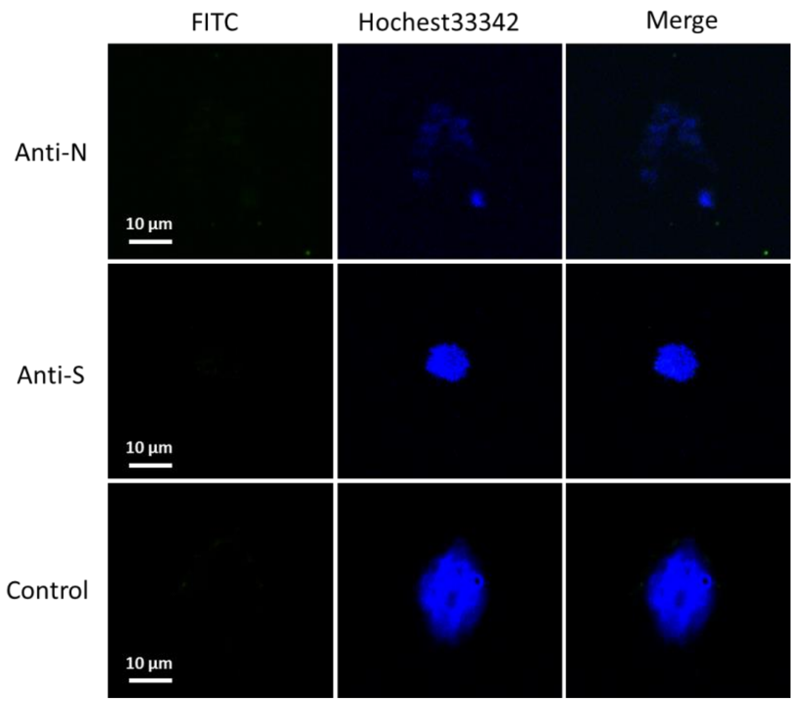

Fig S.1 Fluorescence microscopy results cells recovered from the right eye.

We observed several cells from Glaucoma affected eye positive for SARS-CoV-2 S and N proteins. The main figures we added into main document other results are shown in Fig S.2.

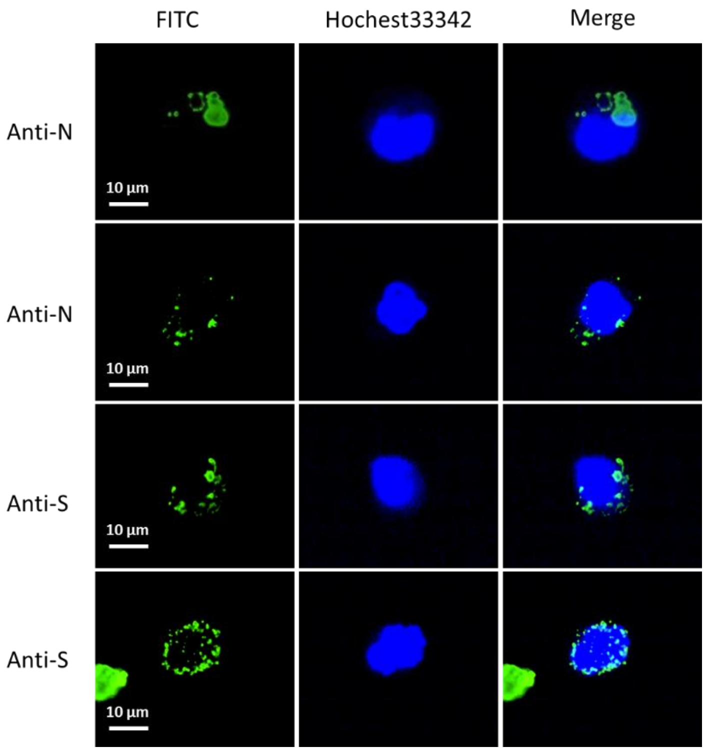

Fig.S.2 Cells from glaucoma affected eye positive for SARS-CoV-2 S and N proteins

3.2.3 Plaque reduction neutralization test (PRNT)

The neutralizing activities of Patient's serum samples were performed by plaque reduction neutralization test (PRNT). Briefly, approximately 50 PFU of SARS-CoV-2 was pre-incubated with 2-fold serial dilutions of the heat-inactivated sera (starting at 1:10 dilution) for 1 h at 37℃ and then the mixture was added to Vero-E6 cell monolayers in 24-well plates and removed after 1 h of incubation followed quantifying virus by plaque assay as described above. Neutralizing antibody titers (PRNT50) were determined to be the highest serial dilutions for which the virus plaque count was reduced by 50% compared with the control.
